# Genetic Analysis of HIV-1 Integrase Sequences from Treatment Naive Individuals in Northeastern South Africa

**DOI:** 10.3390/ijms14035013

**Published:** 2013-03-01

**Authors:** Pascal Obong Bessong, Julius Nwobegahay

**Affiliations:** HIV/AIDS & Global Health Research Programme, University of Venda, Thohoyandou 0950, South Africa; E-Mail: nwobegahay@yahoo.com

**Keywords:** HIV-1 integrase, raltegravir genetic resistance, polymorphisms, viral recombinants, northeastern South Africa

## Abstract

Raltegravir, an integrase inhibitor, is not a component of the current South African antiretroviral treatment guidelines, but it could be introduced in the near future as cases of virological failures from current treatment regimens begin to occur. The aim of this study was to analyze the complete HIV integrase gene obtained from individuals at two treatment sites in northeastern South Africa for the presence of Raltegravir associated drug resistant mutations and viral subtypes based on the integrase gene. Examination for mutations against other integrase inhibitors, such as Elvitegravir and Dolutegravir, was also done. Viruses from 127 treatment naive individuals were analyzed. Genetic drug resistance mutations were determined using the Stanford HIV Drug Resistance Interpretation program and the International AIDS society-USA guidelines. Viral subtyping was done by phylogenetic analysis, and recombinants were determined using the REGA, jpHMM and RIP tools. No major resistance mutations were detected. However, 7% of the sequences had minor mutations and polymorphisms. The majority (99%) of the viruses were HIV-1 C. Recombination analysis showed that the polymerase gene of one virus was likely composed of HIV-1 subtype A1 and C sequences. The present study indicates that Raltegravir, Elvitegravir and Dolutegravir resistant mutations may be absent in the study communities and further indicates the presence of recombinant viruses in northeastern South Africa.

## 1. Introduction

The use of antiretrovirals (ARV) inevitably leads to the emergence of resistant viruses and subsequent treatment failure. The availability of new drug classes, such as integrase inhibitors, provides the possibility of alternative drug combinations, thereby offering more treatment options [1].

Integrase (IN) is an essential viral enzyme, comprising 288 amino acids encoded by the 3′-end of the HIV polymerase gene. Integrase catalyzes the chromosomal integration of newly synthesized double-stranded DNA into the host genomic DNA. It also plays a role in stabilizing a pre-integration complex (PIC), which consists of the 3′-end processed genome, and one or more cellular co-factors involved in nuclear transfer of the PIC [2]. HIV IN comprises three functional domains: the *N*-terminal domain (NTD), which encompasses amino acids 1–49 and contains a histidine-histidine-cysteine-cysteine (HHCC) motif that coordinates zinc binding, the catalytic core domain (CCD), which encompasses amino acids 50–212 and contains the catalytic triad D64, D116 and E152, known as the DDE motif, and the *C*-terminal domain (CTD), which encompasses amino acids 213–288 and is involved in host DNA binding. There is no human homologue of IN, and so, HIV IN represents a rational and important target for halting viral replication and preventing AIDS [3]. South Africa has one of the highest HIV/AIDS epidemics in the world. According to the South African National HIV and Syphilis Sentinel survey of 2010, the estimated national prevalence was 25%, and the Limpopo Province (northeastern South Africa) had a prevalence of 21.4% [4].

The integrase inhibitor Raltegravir was approved by the US Food and Drug Administration for use in patients failing treatment due to drug resistance. Raltegravir in combination with an optimized background regimen significantly improved the viral load and CD4^+^ cell count at week 24 in a phase III clinical trial [5]. Its use is still limited to treatment-experienced patients and subjects with drug resistant virus, although it can be used in a first line treatment regimen for patients with intolerance to some antiretrovirals [6].

Eight years after the introduction of antiretrovirals in public health facilities in South Africa, the development of resistance and treatment failure has begun to emerge [7–9]. Integrase inhibitors (Raltegravir, Elvitegravir and Dolutegravir) may be introduced in South Africa in the near future as components of salvage therapy. Despite this, only a few studies in South Africa have examined the IN gene in terms of viral genetic variability and resistance mutational patterns [7,10]. No data is available from northeastern South Africa, where HIV prevalence is relatively high, and with the possibility that viruses may differ in their sensitivity to integrase inhibitors [11], it is important to provide sequence data on drug targets in the evolving HIV genetic landscape.

## 2. Results and Discussion

### 2.1. Subjects’ Demographics and Resistance Mutations

A total of 127 HIV positive individuals were sequentially recruited. The mean age was 43.5 years (range 18–69 years). Two thirds of the participants were females. Eighty one percent of the study subjects were single and 19% were married. The most important risk factor for HIV transmission was sexual intercourse (94.5%). In 88.2% of the participants, the most probable place of HIV infection was South Africa. Thirty-three percent of the study subjects thought they could have been infected in 2008, while 67% estimated that they were infected between 2000 and 2007. Raltegravir inhibits the function of integrase by inhibiting its strand transfer activity. However, data on the patterns of resistance selection is still emerging [6]. The prevalence of drug resistance is low in most developing countries,, but with the expanding access to therapy, resistance development and selection and subsequent treatment failure are expected to occur over time.

In the present study, expected PCR products were obtained for 120/127 (94.5%) of the subjects. Reliable complete HIV IN nucleotide sequences were obtained for 89 viruses (74%) on which genetic subtyping and drug resistance analysis were performed. The detected mutations, nucleotide substitutions and their potential significance are shown in Table 1. No major Raltegravir, Elvitegravir and Dolutegravir resistance mutations were observed in the study population. However, minor resistance mutations (L74M, Q95K) and polymorphisms (Q95P, E157K, I203M and R263S) for Raltegravir were detected in six different subjects (08MB73ZA, 8BBVCT27ZA, 08MB33ZA, 08BBVCT49ZA, 08BBVCT38ZA and 08MB34ZA).

The absence of major Raltegravir resistance mutations have been recently reported from the Gauteng region of South Africa and in other parts of the world. For example, Fish *et al*. [10] observed polymorphisms among 73 viruses obtained from HIV-1 infected South African patients, and a similar scenario was reported from Mozambique [12]. Arruda *et al*. [13] also reported the absence of resistance mutations among 76 HIV-1 infected integrase inhibitor-naive patients in Sao Paulo, Brazil. The potential impact of the polymorphisms observed in the current study and reported elsewhere is not yet fully understood. The full relevance of these mutations in clinical practice has yet to be defined in light of the lack of a sufficient number of long-term treatment follow-up studies [3,14]. It is important that appropriate molecular models be designed to enable phenotypic characterization of these polymorphisms.

### 2.2. HIV Integrase Genotypes

**Figure 1:**
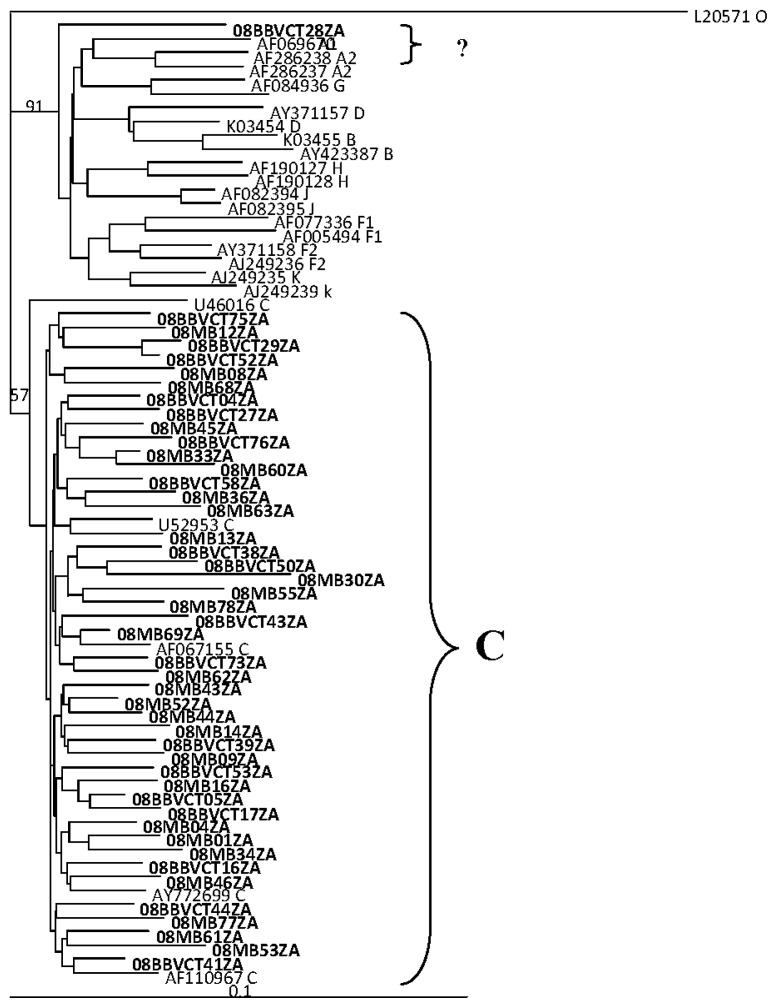
Phylogenetic analysis of HIV IN nucleotide sequences from drug naive patients from northeastern South Africa. The tree was generated by the neighbor-joining method and shows the test sequences (in bold face) clustering and intermingling with reference HIV-1 subtype C integrase sequences previously documented from Southern Africa and elsewhere. Virus 08BBVCT28ZA could not be definitely assigned.

Phlyogenetic analysis of the sequences showed that 88/89 (99%) of the viruses were HIV-1 subtype C without clonal providence. One virus (08BBVCT28ZA) could not be assigned by phylogenetic analysis (See Figure 1). The sequence was assigned as subtype F in the Stanford drug resistance analysis.

**Figure 2:**
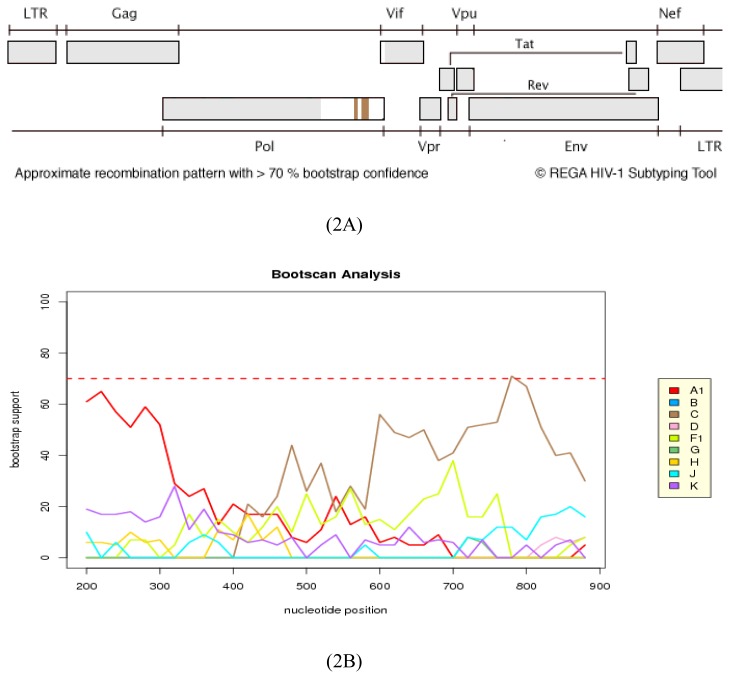
Recombination analysis of 08BBVCT28ZA IN gene. In 2**A** & 2**B**, sequence assignment and bootscanning with REGA was done to determine possible recombination in the integrase gene (bootscanning, window size of 400 and step size 20). 2**A** shows the sequence as subtype C; 2**B** shows the bootscanning analysis of the recombination breakpoints with a bootstrap value of more than 70%. The results show that the sequence has a mosaic structure consisting of HIV-1 A1 alternating with a subtype C sequence, suggesting that the virus is a recombinant on the IN gene.

As a result of the ambiguous assignment of 08BBVCT28ZA by phylogenetic analysis, the complete integrase gene (864 bp) and a sequence comprising the complete protease and partial reverse transcriptase (1127 bp, HXB2 position: 2253–3379) was further analyzed for recombination using REGA. REGA assigned the integrase sequence to A1 with unclassified regions, although with a less than 70% bootstrap support. Nevertheless, it is worthwhile to note that the IN sequence was assigned as F, F1/F2 and F1 in the Stanford, RIP and jpHMM analyses tools, respectively (data not shown) (See Figure 2).

**Figure 3:**
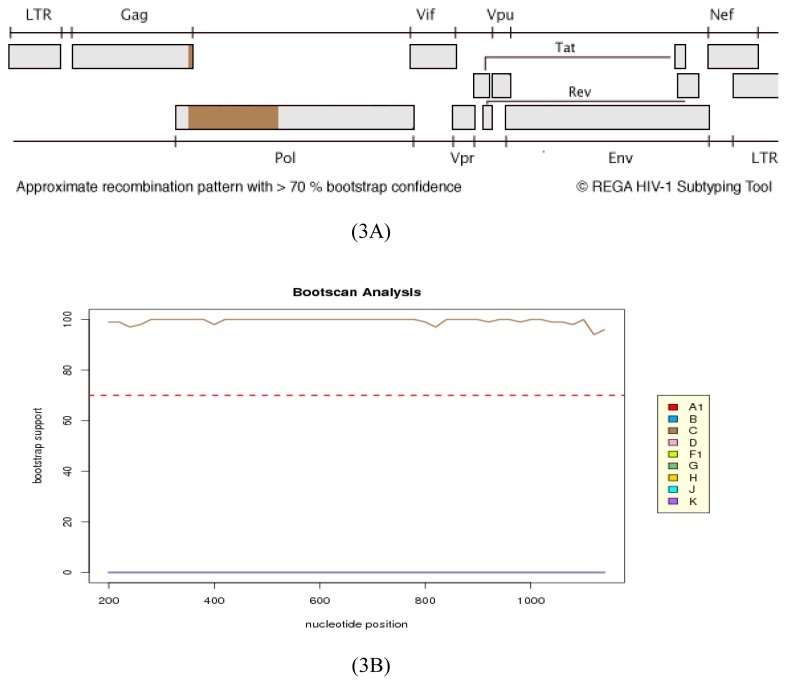
Recombination analysis of 08BBVCT28ZA protease/reverse transcriptase sequence. In 3**A** & 3**B**, sequence assignment and bootscanning of the sequence (1127 bp, HXB2:2253–3379) using REGA shows that the sequence is HIV-1 subtype C with a high bootstrap support.

On the other hand, the protease/reverse transcriptase sequence was assigned to HIV-1 subtype C by REGA analysis with a high bootstrap value (Figure 3).

All the integrase sequences, except one, were HIV-1 subtype C, an observation in line with other studies that have shown that the epidemic in northeastern South Africa is highly dominated by HIV-1 subtype C viruses [15,16]. However, the polymerase sequence of one virus as indicated had a genome comprising sequences of HIV-1 subtypes A1 and C. Although this analysis was restricted to the polymerase gene, the observation suggests the introduction of viruses with novel genomes in the study area. It is worthwhile to note that two A1/C recombinants in the integrase gene have been previously reported in two individuals in a study population in Johannesburg (Gauteng province), South Africa [17].

### 2.3. Analysis of the Functional Domains of HIV-1 IN Gene

**Figure 4:**
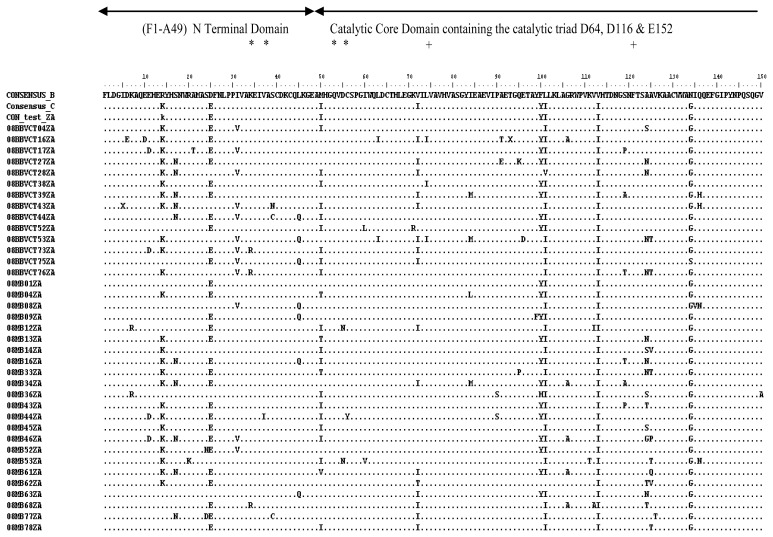
Alignment of amino acid residues of HIV-1 subtype C (residues 1–150). The consensus was generated from 37 viruses. The global subtype B and C consensuses were obtained also from HIV sequence database. The entire consensus sequences were aligned using BioEdit software. The conserved residues in the HHCC motif (*) and the DDE (+) are noted. All positions that agree with the global subtype B consensus sequence are denoted by a dot (.); while ambiguous amino acids are denoted by an X. The test consensus was identical to the global subtype C consensus, except at two positions (V72I and R269K). It differed from the global subtype B consensus at 10 positions (R14K, D25E, M50I, F100Y, L101I, V113I, N134G, E167D, T218I and D278A).

**Figure 5:**
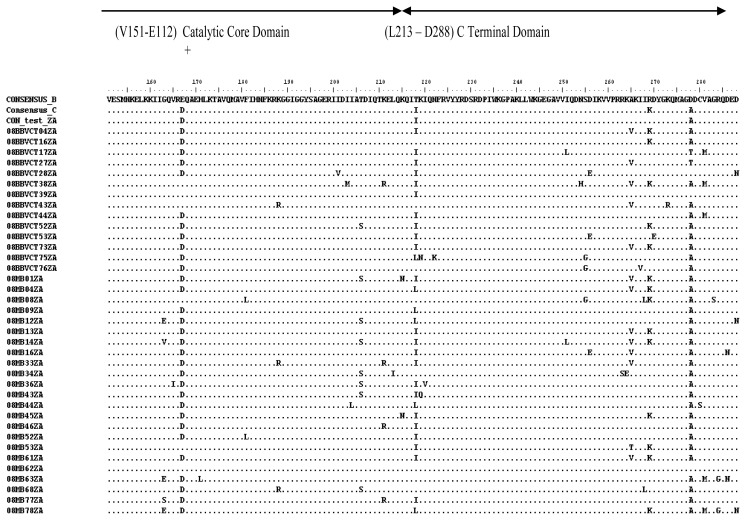
Alignment of amino acid residues of HIV-1 subtype C (residues 151–288). The test consensus was generated from 37 viruses. The global subtype B and C consensus sequences were obtained also from HIV sequence database. The conserved residue E, which is an integral part of the DDE motif is noted (+). All positions that agree with the global subtype B consensus sequence are denoted by a dot (.); while ambiguous amino acids are denoted by an X.

There was little genetic variability in the IN gene as expected, with a mean genetic distance range of 0.0124 to 0.1004. Considering the complete predicted amino acid sequence (288 amino acid residues), sequence alignment showed that the consensus of the test viruses was identical to the global subtype C consensus, except at two positions (V72I and R269K). It differed from the global subtype B consensus at 10 positions (R14K, D25E, M50I, F100Y, L101I, V113I, N134G, E167D, T218I and D278A) (Figures 4 and 5). The *N*-terminal and catalytic core domains were identical to the global subtype B consensus. However, the *C*-terminal domain differed at positions T218I and D278A. There were no insertions or deletions. The integrase nucleotide sequences reported here have been submitted to GenBank with the following accession numbers: HM569270–HM569358.

On the whole, 11 of the 288 (3.8%) amino acid sequences had substitutions. The three functional domains of integrase are generally conserved. The NTD, which encompasses amino acids 1–49 and contains a HHCC motif, was conserved, and the two substitutions observed (T218I and D278A) were not in the HHCC motif. The HHCC motif coordinates zinc binding and helps in protein stability. The CCD, which encompasses amino acids 51–212 and contains the catalytic DDE motif, was also conserved, and the six substitutions observed are not within the DDE motif. The CTD, which encompasses amino acids 213–288, was generally conserved. Two substitutions (T218I and D278A) occurred in this region, representing a variability of 2.2% (2/89). These substitutions are conservative changes, which do not seem to have any effect on integrase activity [18,19]. All the three domains are important in protein stability, multimerization, catalytic activity, binding with DNA and the human cellular co-factor LEDGF/p75 [3].

## 3. Experimental Section

### 3.1. Ethical Considerations and Study Sites

Approval of the study protocol was obtained from the Health, Safety and Research Ethics Committee of the University of Venda, South Africa. Permission was obtained from the Limpopo Provincial Department of Health and authorities of the HIV/AIDS Prevention Group in Bela-Bela and Mankweng Hospital. Signed informed consent was obtained from all study participants prior to sample and demographic data collection. Study subjects were recruited from the Phela O’ Phedishe (POP) HIV clinic in Mankweng Hospital and the Bela-Bela HIV/AIDS Wellness Clinic in Bela-Bela. The geographical characteristics of these treatment sites have been described [20].

### 3.2. Study Population, Sample Collection and Plasma Preparation

Individuals without prior exposure to ARV visiting the voluntary counseling and testing facilities of the Bela-Bela Wellness and POP clinics and who tested positive for HIV were recruited sequentially between February 2008 and December 2008. Five ml of venous blood was collected into EDTA vacutainer tubes and spun at 3000 rpm for 3 min. Plasma was aspirated aseptically and stored at −80 °C for subsequent use. Demographic data, such as age, sex, place of residence, probable date and place of infection and marital status was obtained by questionnaire administration. Viral load and CD4 cell count data were not available.

### 3.3. Viral RNA Isolation, RT-PCR and Nested PCR

Viral RNA was isolated using the viral mini RNA kit (Qiagen, Hilden, Germany) and stored at −80 °C until used. A one-tube reverse transcriptase PCR was performed with AMV RT (Roche, Mannheim, Germany) and Taq DNA polymerase (Invitrogen, California, USA), followed by a nested PCR. The first round primers were INFORI (5′-GGA ATC ATT CAA GCA CAA CCA GA-3′; HXB2 location 4059→4081) and INREV-I (5′-TCT CCT GTA TGC AGA CCC CAA TAT-3′; HXB2 location 5244←5267), and the nested primers were HIV+4141 (5′-TCT ACC TGG CAT GGG TAC CA-3′; HXB2 location 4141→4160) and INREVII (5′CCT AGT GGG ATG TGT ACT TCT GA-3′; HXB2 location 5197←5219). The reaction mix (25 μL) comprised 2.5 μL 10× buffer, 2 μM of each primer, 100 μM dNTPs, 0.4 U Taq polymerase, 0.2 U RNAse inhibitor, 0.2 U AMV, 1.5 mm MgCl_2_ and 5 μL RNA. The cycling conditions for the first round were: one cycle of 42 °C for 60 min and one cycle of 95 °C for 3 min. This was followed by 35 cycles of 95 °C for 1 min, 54 °C for 1 min and 72 °C for 2 min and a final extension at 72 °C for 10 min. This generated a product of approximately 1078 bp (HXB2 location 4230–5093) of the complete HIV-1 integrase gene. Five microliters of the first round reaction was used as template in the nested PCR. The reaction mix (50 μL) comprised 5 μL 10× buffer, 2 μM of each primer, 100 μM dNTPs, 0.4 U Taq polymerase and 1.5 mm MgCl_2_. The cycling conditions were: one cycle of 95 °C for 3 min, 35 cycles of 95 °C for 1 min and 54 °C for 1 min and 72 °C for 2 min, followed by a final extension at 72 °C for 10 min.

The PCR products were verified for expected size by electrophoresis of 1% agarose gel stained with ethidium bromide. Amplicons were purified with QIAquick PCR purification kit (Qiagen, Hilden, Germany), and direct population-based sequencing was performed on both strands with the Big Dye Terminator v3.0 kit on ABI Prism 377 (Applied Biosystems, California, CA, USA) using Taq DNA polymerase. Generated nucleotide sequences were edited manually using SeqMan II version 7 (DNASTAR, Wisconsin, WI, USA).

### 3.4. Genetic Subtyping and Resistance Analyses

Viral subtyping was done by phylogenetic analysis. Nucleotide sequences of test viruses were aligned using Clustal X with representative subtype reference sequences (group M subtypes A–D, F–H, J and K) obtained from GenBank. Previously described integrase sequences from Southern Africa were included in the analysis. Neighbor joining phylogenetic trees were generated with the PHYLIP program. Trees were rooted with HIV-1 group O reference strain (L20571). The reliability of sequence clustering was assessed by a bootstrapping of 1000 replicates. Recombination analysis was done using REGA, Recombination Identification Program (RIP) and jumping Profile Hidden Markov Model (jpHMM) tools [21,22]. Drug resistance mutations were determined according to the Stanford HIV Drug Resistance Interpretation Algorithm [23] and the International AIDS Society-USA Guidelines [24].

### 3.5. Analysis of the Functional Domains of HIV-1 Integrase Gene

Integrase gene predicted amino acids were aligned using the BioEdit program [25]. Briefly, consensus amino acid sequences for IN were created and then compared to the global subtype C and the global subtype B consensus sequences obtained from GenBank (year 2000). The functional domains (F1–A49, M50–E112 and L213–D288) on the test consensus were then compared with those of the global C and global B, in order to determine conserved and substituted amino acid residues. The mean genetic distances among the sequences were determined by the Kimura 2-parameter model.

## 4. Conclusions

The present study has provided baseline sequence data on HIV-1 subtype C integrase from northeastern South Africa and supplements the scanty data on integrase genetic diversity in South Africa. The data also suggests the absence of major Raltegravir, Elvitegravir and Dolutegravir mutations in the study population. In addition, the results point to the need for regular molecular epidemiology studies to detect genetic changes in infecting viruses relevant for prevention and treatment strategies.

## Figures and Tables

**Table 1:** Drug resistance associated mutations, frequency and coding nucleotides in HIV-1 subtype C integrase sequences from northeastern South Africa.

Sample code	Integrase mutation	Frequency	Coding nucleotides		Comment

			Wild type	mutant	
08BBVCT73ZA	L74M	1/89 (1.1%)	CTG	ATG	Polymorphism (Stanford); minor mutation involved in the Q148H/K/R pathway to Raltegravir resistance (IAS).
08BBVCT27ZA	Q95K	1/89 (1.1%)	CAA	AAA	Minor mutation to Raltegravir (Stanford); not documented in IAS algorithm
08MB33ZA	Q95P	1/89 (1.1%)	CAA	CCA	Unusual mutation (Stanford); not documented in IAS algorithm.
08BBVCT49ZA	E157K	1/89 (1.1%)	GAA	AAA	Unusual mutation (Stanford); mutation not documented in IAS algorithm.
08BBVCT38ZA	I203M	1/89 (1.1%)	ATA	ATG	Polymorphism; but not documented in IAS algorithm.
08MB34Z	R263S	1/89 (1.1%)	AGG	AGC	Unusual mutation by Stanford; but not documented in IAS algorithm.
